# Clinical protein science developments for patient monitoring in hospital central laboratories

**DOI:** 10.1186/s40169-016-0127-0

**Published:** 2016-12-19

**Authors:** Johan Malm, György Marko-Varga

**Affiliations:** 1 Department of Translational Medicine, Section for Clinical Chemistry, Lund University, Skåne University Hospital Malmö, 205 02 Malmö, Sweden; 2 Center of Excellence in Biological and Medical Mass Spectrometry, Lund University, BMC D13, 22100 Lund, Sweden; 3 Clinical Protein Science & Imaging, Biomedical Center, Department of Biomedical Engineering, Lund University, BMC D13, 221 84 Lund, Sweden; 4 First Department of Surgery, Tokyo Medical University, 6-7-1 Nishishinjiku Shinjiku-ku, Tokyo, 160-0023 Japan

**Keywords:** Biomarker, Proteomics, Assay, Mass spectrometry

## Abstract

Patient care relies heavily on standardized tests performed in hospital laboratories, typically including clinical chemistry, pathology and microbiology. With the introduction of personalized medicine tremendous efforts have been made to identify new biomarkers of disease with various omics technologies, often including mass spectrometry. In order to validate new biomarkers and perform clinical studies high quality biobank samples are of key importance. In this editorial different aspects of mass spectrometry in future personalized medicine are discussed.

## Editorial

Every day thousands and thousands of human tissue samples are sent to hospital laboratories for analysis. The results constitute the major source of diagnostic, prognostic and disease monitoring information on patient health, and forms the basis for the decision making by clinicians. The healthcare sector relies on standardized assays that can be made comparable in both Lund, Sweden, as well as, e.g. in Hyderabad, India. Today in our globalized healthcare driven society, the instrumental companies can ship, and implement any assay in a laboratory in any continent. Assay standards are made available that allows results from different laboratories to be compared, thus building a joint world wide resource for patient care. The actual measurement and its analytical characteristics is the key for patient diagnosis, and disease status determination. Compared to the total cost of patient care, laboratory based information is often relatively cheap. Tissue samples, in most cases blood, are sent to the hospital laboratory not only from different hospital wards but also from e.g. primary care units, private practices, and occupational health clinics. Already today, and even more tomorrow, a substantial number of these samples will also be transferred to and stored in *large scale Biobanks* [1]. Some of the most common routine tests in clinical chemistry laboratories are; CBC (complete blood count), electrolytes, *hormones, coagulation parameters, disease specific protein biomarkers and plasma biomarker proteins*. In clinical microbiology laboratories the quantitatively dominating tests, performed on e.g. urine, blood and swab cultures, are ordered to identify the microorganism causing infection. These tests are often carried out in conjunction with susceptibility testing to determine specific antibiotics that will inhibit the growth of the microbe. In addition, the clinical pathology laboratories process tissue samples subsequently being subject to microscopic evaluation for diagnosing disease. Hospital laboratories have used mass spectrometry for many years, mainly for the analysis of therapeutic drugs and drugs of abuse, but lately also as multiplex assay analyzers for endogenous markers. As *mass spectrometer platforms* have improved over time, with an increase in both sensitivity and resolving power, and with an easier to use approach, there has been increasing expectations that many of the routine hospital lab analyses would be performed using mass spectrometry technology, instead of e.g. immunochemical methods. Not only mass spectrometry but also other ‘omics tools’ have become more widely and affordably available, and these new technologies form the basis for an accurate and versatile monitoring of personalized medicine treatments.

During the last decades tremendous efforts have been made to identify and introduce novel biomarkers, especially cancer biomarkers, in clinical medicine. These biomarkers, whether biomarkers for cancer or other diseases, may be of varying molecular types, such as; *proteins, DNA, RNA, and metabolites*, are found in e.g. blood, urine or other tissue samples. In spite of improved technology capabilities, the introduction of what is often called “precision medicine” or “personalized medicine” in hospital routine laboratories has been slower than expected, mainly for two reasons;

- the degree of automation
- the interpretation of data.

Most of today’s routine analyses are fairly cheap, highly automated and have short turn-around times. The resulting outcome is in most cases, easily interpreted. Mass spectrometry on the other hand has traditionally been labor intensive and the interpretation of the resulting, often, complex data a bottleneck. However, the different ‘omics tools’, and in particular mass spectrometry, have become faster and more easy to use. The instrument purchase still remains the major cost, while the running costs still are low in comparison to ELISA platforms. With dedicated assays, the data can be easily handled as relative or absolute measures in patient samples.

One of the most widely used omics technology platforms is mass spectrometry based detection of *somatic gene mutations* using the Sequenome *DNA mass-array technology*. Here matrix-assisted laser desorption ionization time of flight (MALDI-TOF) technology has been adapted to high throughput screening. One of the most recent, ‘new’ fields using mass spectrometry in routine diagnostics is the identification and typing of bacteria, especially after the approval by FDA of two MALDI-TOF–MS based platforms [2, 3]. By the use of MALDI mass spectrometry technology many cultivable microorganism in clinical samples can be identified, and mass spectrometry based diagnostics can be used for rapid hospital laboratory diagnosis of common organisms including those that are difficult to culture. Fast and specific identification of microorganisms will be even more important in the future due to the increasing problems with antibiotic resistance. In patients with sepsis, one of the leading causes of death in non-coronary intensive care units, early treatment is of utmost importance and often impacts patient outcome. Recently, clinical reports position sepsis as a major challenge to the healthcare sector and to our society. Sepsis patients constitute a healthcare problem with the same magnitude as a major part of cancer patients per year, partly due antibiotic resistance. Consequently, rapid identification of the blood stream infections, and the identity of the particular microorganism causing the disease is a prerequisite for initiating adequate antibiotic therapy. There are dedicated kits for *Sepsis*; The *MALDI Biotyper Sepsityper®*, which enable a proteomic profile to be established, with high resolution mass spectrometry that applies sequence libraries for a positive identification of various bacteria directly from positive blood cultures. Another example of proteomics based *identification of microorganisms* is the identification of mycobacteria using the “Mycobacteria Library”, which currently can manage the positive identity of 159 out of the 169 known species of today, applying high end bioinformatics software. Following the regulatory guidelines, the sample is submitted to the Biotyper mass spectrometry platform for screening and identification of blood samples, the diagnosis is established in less than 30 min after detection of a positive blood culture. Typically, 96 samples can be fully analyzed in one cycle period using MALDI Mass Spectrometry technology as a high throughput platform [4].

In the hospital pathology laboratory the histopathological diagnosis has long been performed by haematoxylin and eosin stained tissue samples. This method is now routinely complemented by immunohistochemistry and often also by electron microscopy and molecular techniques. During the last two decades *mass spectrometry imaging* (MSI) has been used for direct analysis of tissue sections. Especially MALDI links molecular analysis to traditional histology. MSI may add information to the histopathological analysis, confirm the diagnosis and aid in the choice of treatment [5]. Our knowledge of diseases is being advanced in large population-based studies, and the use of data from these epidemiological resources will highly impact our ability to both model and solve disease-related questions in the future. Clinical samples represent a valuable resource not only in routine patient care, but also for investigating the many factors that drive human biology in various disease settings. Biomarkers present in clinical samples bring important read-outs of health and disease. When this data is combined with other types of patient information, it can aid decision making by physicians in applying the optimal patient treatment procedure. Improved analytical performance characteristics requires high quality samples for reliable results. There are many steps in sample processing, storage, and management that need to be understood and pursued in R&D operations. These work flows and integrations provide not only preservation of the desired analytes in the sample, but are also a prerequisite to comply with the moral and legal framework required for human subject protection irrespective of where the samples have been collected. Laboratory medicine should make a great effort in todays community work to align and standardize the methodology of endogenous biomolecule determination. It should include sample storage performed in biobanks, and the necessary frameworks of human subject protection that includes informed consent, and institutional review of the studies being performed.

Global healthcare is under pressure to meet the demands from patients to improve efficiency, especially in treatment of cancer diseases. Societies have difficulties in managing an increasing burden of healthcare costs. New drug characterization assays are central in providing evidence to the specificity and selectivity of drugs. Targeted drug development requires mode of drug action mechanistic studies in order to get approval from the FDA. Large scale protein sequencing studies are undertaken to discover different molecular protein forms, i.e. “Proteoforms” that can be linked to disease, and treatment processes, both as drug targets as well as biomarkers. Multi-dimensional liquid chromatography (MDLC) separation systems within automated platforms are built with on-line as well as off-line liquid phase systems, applied in proteo-genomic studies applying both cation-exchange and anion exchange mechanisms. A recent Cell paper by the ISB-team in Seattle [6], describes the SRM-Atlas, a milestone achievement, which is a compendium of assays that enables quantification of 99.7% of the 20,277 annotated human proteins. This includes a selection of splice variants, non-synonymous SNPs, and N-glycosylated proteins, by selected reaction monitoring (SRM). This resource enables the accurate detection and quantification of any known or predicted human protein. We envision that protein quantification by MS-platforms will constitute a paradigm shift in hospital laboratories worldwide; targeted analysis will be made by *selected reaction monitoring “SRM” assays*. These SRM assays are also easy to multiplex, whereby a number of 10–100 protein biomarkers can be analysed in a single assay cycle. There are already instruments in place in hospitals being tested as pilot units in a high throughput fashion for the quantitative analysis of proteins. The ageing population world wide will generate more patients who expect health services to provide fast and accurate diagnostic tools—mass spectrometry based assays can be expected to play a major role in tomorrows hospital laboratories. But not only for analysis of disease—probably also for analysis of health. There is a trend where measuring subjective well-being and people’s quality of life is fundamental when assessing the progress of societies. These measurements can be expected to include screening in central hospital laboratories, e.g. in the analysis of blood plasma, the most easily accessible human sample.

In conclusion, we envisage a future where a further technology development positions mass spectrometry as a key technology in novel and highly automated analytical platforms in hospital routine laboratories, that will be used in diagnosis, prognosis and monitoring of disease—and health. The developments are seen within current applications of personalized medicine strategies, by matching specific disease genotypes and phenotypes with specific pharmaceutical drug treatment. The expansion of this treatment paradigm into other diseases will further lead to new opportunities within the diagnosis and treatment of diseases, particularly for “*Precision Medicine*”.
